# Clinical Characteristics and Prognosis in Spontaneous Isolated Abdominal Aortic Dissection Based on the Dissection Length

**DOI:** 10.3390/jcm14165849

**Published:** 2025-08-19

**Authors:** Saddam Shaiea, Xingwei He, Hussen Mansai, Fatima Aldali, Abdulwahab Hashem, Ye Heng, Hesong Zeng

**Affiliations:** 1 Department of Cardiology, Tongji Hospital, Tongji Medical College, Huazhong University of Science & Technology, Wuhan 430030, China; 2 Department of Internal Medicine, Mayo Clinic Health System, Mankato, MN 56001, USA; 3 Department of Rehabilitation Medicine, Tongji Hospital, Tongji Medical College, Huazhong University of Science & Technology, Wuhan 430030, China; 4 Department of Cardiology, Union Hospital, Tongji Medical College, Huazhong University of Science & Technology, Wuhan 430022, China

**Keywords:** spontaneous isolated abdominal aortic dissection, CT angiography, arteriosclerosis, endovascular repair, outcome analysis

## Abstract

**Objective**: The purpose of this study was to report the clinical characteristics and prognosis of spontaneous isolated abdominal aortic dissection (SIAAD) based on the dissection length. **Methods**: Between March 2012 and September 2023, 159 of 7572 patients with aortic dissection were diagnosed with SIAAD and enrolled in the retrospective study. We proposed a new morphologic classification: extensive SIAAD (e-SIAAD) and focal SIAAD (f-SIAAD), based on whether the dissection length exceeds 50 mm or not. The clinical baseline, computed tomography angiography (CTA) findings, and long-term follow-up of the two types were compared. **Results**: SIAAD prevalence was 2.1%. Patients with f-SIAAD were significantly older (63.74 ± 10.97 vs. 50.70 ± 10.10 years, *p* < 0.001), had more atherosclerosis risk factors, arteriosclerosis, and penetrating aortic ulcers compared to e-SIAAD patients. Conversely, e-SIAAD presented more acutely (72.97% vs. 34.12%, *p* = 0.001), exhibited more frequent symptoms (85.14% vs. 61.18%, *p* = 0.0037), larger dissection diameters (31.89 ± 10.99 vs. 24.41 ± 11.28 mm, *p* = 0.001), and greater involvement of the renal and iliac arteries. Treatment involved medical management (30%), endovascular repair (65%), or surgery (2.5%), without significant differences between groups. In-hospital mortality was higher in f-SIAAD (six deaths vs. one in e-SIAAD). During median follow-up of 48 months (range, 6–148 months), mortality was higher in f-SIAAD (70% vs. 90% estimated 10-year survival). **Conclusions**: SIAAD classification by dissection length revealed significant differences in clinical presentation, CTA characteristics, and prognosis. Focal dissections correlated with advanced age, severe arteriosclerosis, and poorer long-term outcomes, emphasizing the need for tailored management approaches.

## 1. Introduction

Spontaneous isolated abdominal aorta dissection (SIAAD) is an infrequent and potentially fatal condition, accounting for 1% to 4% of all aortic dissections [1,2,3]. In SIAAD, the dissection flap is confined to the abdominal aorta, with no extension into the thoracic aorta. This distinguishes it from the classic DeBakey/Stanford classifications of aortic dissection, which primarily describe thoracic involvement. The first SIAAD was reported nearly two centuries ago, in 1822 [4]. Yet, even after 185 years since that report, the epidemiology, pathophysiology, and optimal treatment strategy remain unclear. Risk factors include hypertension, atherosclerosis, and connective tissue disorders.

Most published experience consists of individual case reports and small case series, which limit our understanding of this rare entity [5,6,7]. In those studies, the classification of SIAAD was primarily based on the anatomical relationship between the location of the primary entry tear and the orifices of abdominal vessels (such as suprarenal and infrarenal) to guide endovascular repair and surgery [8]. However, little research has focused on the classification of the morphological characteristics of SIAAD itself. Some SIAADs, like thoracic aortic dissection, cause a relatively long dissection; yet other SIAADs cause a quite short dissection. This may suggest different pathological features between the extensive (long) and focal (short) SIAADs.

Accordingly, the aim of this retrospective study was to explore and compare the risk factors, clinical characteristics, treatment modalities, and long-term follow-up results between patients with the extensive and focal SIAAD.

## 2. Methods

This retrospective study was conducted at Tongji Hospital, a single high-volume tertiary care center in Wuhan, China, over a 12-year period from March 2012 to December 2023. Of the 7572 patients diagnosed with aortic dissection (AD) during this time, 159 cases met the criteria for SIAAD. SIAAD was defined as any AD exclusively involving the aorta below the diaphragm on spiral computed tomography angiography (CTA) and confirmed by at least two experienced interventional cardiologists. Patients with intramural hematoma (IMH), thoracic AD, and iatrogenic and traumatic SIAAD were excluded. Residual abdominal AD after endovascular repair for thoracoabdominal AD was also excluded.

The Ethics Committee of Tongji Hospital approved this retrospective observational study, Huazhong University of Science and Technology (TJ-IRB20211102). All aspects of the study comply with the principles outlined in the Declaration of Helsinki.

All patients were diagnosed by computed tomography angiography (CTA) using a 64-detector row scanner (LightSpeed VCT; GE Healthcare, Milwaukee, WI, USA). A non-ionic contrast medium containing 300 mg iodine/mL iopromide (Ultravist 370^®^; Bayer Healthcare, Berlin, Germany) was administered intravenously in a total volume of 120 mL.

### Data Collection and Classifications

Demographics, cardiovascular risk factors, clinical presentations, aortic CTA features, treatment modality, and in-hospital and long-term outcomes were collected through medical records and via telephone follow-up or mailed questionnaire when necessary.

**Figure 1:**
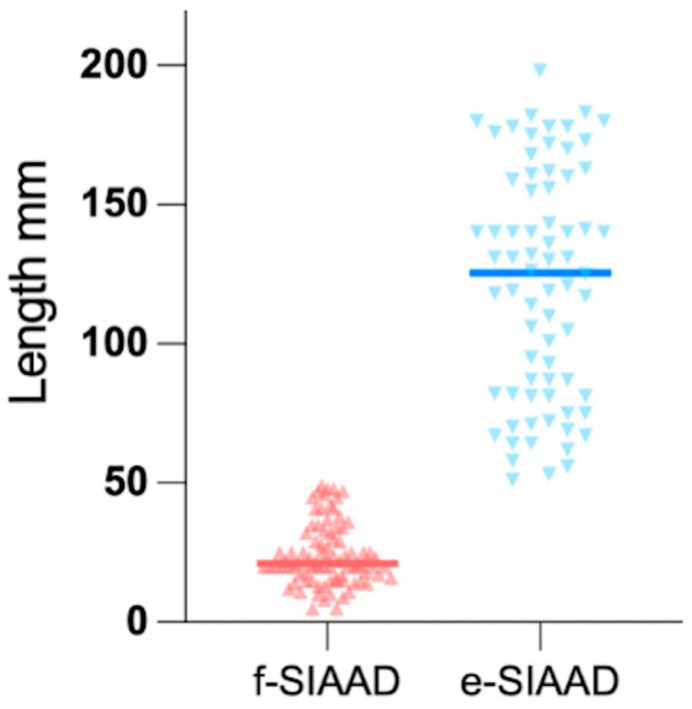
A clear bimodal distribution is evident, with a distinct reduction in frequency near 50 mm, supporting the classification into focal (f-SIAAD, ≤50 mm) and extensive (e-SIAAD, >50 mm) subtypes. The mean dissection length of extensive-SIAAD (118.93 ± 43.65 mm) is significantly greater than that of focal-SIAAD (24.48 ± 10.92 mm; *p* < 0.001). SIAAD, spontaneous isolated abdominal aortic dissection.

To objectively establish the threshold distinguishing extensive (e-SIAAD) from focal SIAAD (f-SIAAD), we analyzed the distribution of dissection lengths (Figure 1). Notably, a clear bimodal distribution was observed, with a natural separation occurring near 50 mm, where a distinct reduction in frequency was evident. For the sake of convenience, we defined the dissection length >50 mm as e-SIAAD (*n* = 74) and that ≤50 mm as f-SIAAD (*n* = 85).

According to the time interval from symptom onset to hospital admission, SIAAD was divided into two groups: acute (≤2 weeks) and chronic (>2 weeks) SIAAD [9]. According to the location of the primary entry site, SIAAD was categorized into three groups: supraceliac (above the celiac artery), paravisceral (between the celiac artery and the lowest renal artery), and infrarenal (below the lowest renal artery) [10].

Other imaging data on aortic CTA (like SIAAD maximum diameter, false lumen thrombosis, aortic arteriosclerosis, abdominal aortic aneurysm [AAA], penetrating aortic ulcer [PAU], and branch artery involvement) were also collected and evaluated. PAU was defined as the presence of a filling defect in the aorta, without the intimal flap and true/false lumens.

## 3. Statistical Analysis

Categorical data were presented as frequency and percentage. Continuous variables were presented as mean ± standard deviation for normally distributed data, or as median with interquartile range (IQR) for non-normally distributed data.

A comparison between the extensive and focal SIAAD was performed using the Fisher exact test for categorical data and the independent Student *t*-test for continuous data. Kaplan–Meier survival analysis was performed for time-to-event data, and differences between survival curves were assessed using the log-rank test.

Statistical analyses were performed with the SPSS software (version 21.0) and GraphPad Prism version 9.5.0 (GraphPad Software, San Diego, CA, USA). A two-sided value of *p* < 0.05 was considered statistically significant.

## 4. Results

### 4.1. Patient Characteristics and Clinical Presentation

The prevalence of SIAAD in this study was 2.1% (159 per 7572 AD patients). The baseline characteristics of SIAAD patients are described in Table 1. The mean age was 57.7 ± 12.4 years (range, 31–86 years), and 119 (74.8%) patients were men. SIAAD patients often had a history of hypertension (112, 70.44%), with a relatively high prevalence of coronary artery disease (56, 35.22%). There were 115 (72.33%) patients with SIAAD-related symptoms on admission. Abdominal pain was the most common symptom (62, 38.99%). A total of 83 (52.2%) of patients were admitted to the hospital in the acute phase of SIAAD. Of 67 (47.8%) chronic SIAAD patients, 24 (36%) had SIAAD-related symptoms; 17 (25%) were asymptomatic; and 26 (39%) patients were admitted to the hospital for other conditions: among these, acute coronary syndrome was slightly the most frequently observed. Notably, less than half of the patients (77, 48.43%) were initially diagnosed with dissection upon admission.

The number ratio of f-SIAAD (*n* = 85) and e-SIAAD (*n* = 74) was close to 1:1. Compared with patients with e-SIAAD, those with f-SIAAD were much older (66 ± 14 vs. 49 ± 13 years, *p* < 0.001) and more likely to have a history of hypertension, diabetes mellitus, coronary artery disease, and chronic renal insufficiency. Moreover, patients with f-SIAAD were more frequently admitted to hospital in chronic phase of SIAAD (56 [66.88%] vs. 20 [27.03%], *p* = 0.001) and had less SIAAD-related symptoms (including chest, abdominal, back, and lumbar pain) (52 [61.18%] vs. 63 [85.14%], *p* = 0.0037). Furthermore, AD as the initial diagnosis on admission was less frequently reported in f-SIAAD than in e-SIAAD (21 [42.71%] vs. 51 [68.92%], *p* = 0.001). There was no statistically significant difference in terms of sex, smoking, and alcohol between the patients with f-SIAAD and e-SIAAD.

### 4.2. CTA Imaging Findings

**Figure 2:**
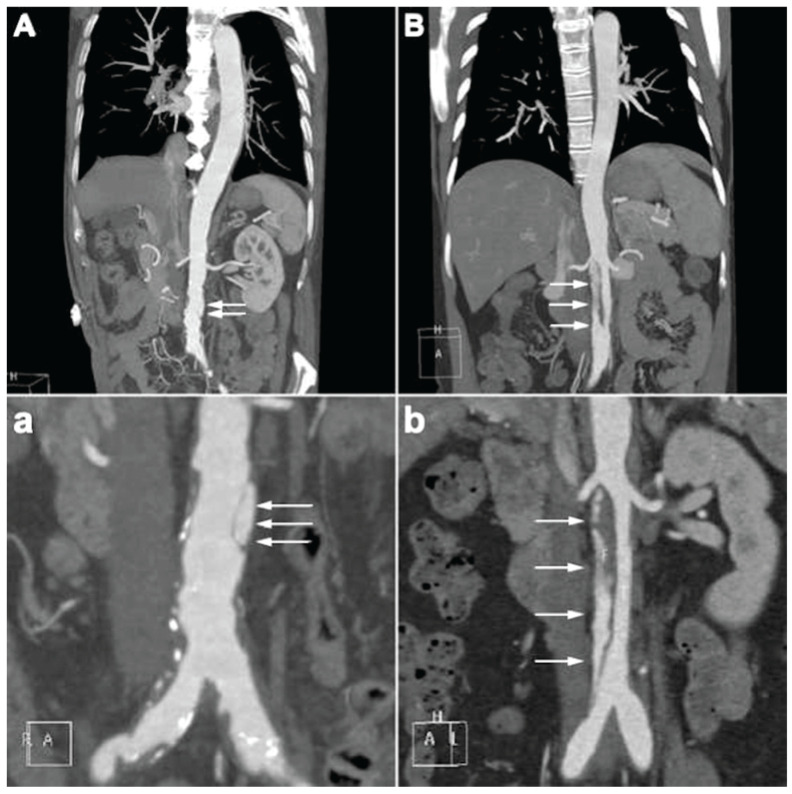
(**A**,**a**) the characteristic of focal-SIAAD on aortic CTA is the presence of a short intimal flap in the abdominal aorta, often with aortic arteriosclerosis. (**B**,**b**) the characteristic of extensive-SIAAD on aortic CTA is the presence of a long intimal flap in the abdominal aorta, normally without apparent aortic arteriosclerosis. Two partial enlarged images of (**A**,**B**) are shown in (**a**,**b**), respectively. White arrows denote the intimal flap and false lumen. CTA, computed tomography angiography; SIAAD, spontaneous isolated abdominal aortic dissection.

The representative CTA images of e-SIAAD and f-SIAAD are presented in Figure 2, and the aortic CTA characteristics are reported in Table 2. Mean dissection length was 68.4 ± 56.3 mm (5–198 mm). Most primary entries (103, 64.78%) were located under the lowest renal artery (infrarenal-SIAAD). The length of dissection was significantly longer in e-SIAAD than that in f-SIAAD (118.93 ± 43.65 vs. 24.48 ± 10.92 mm, *p* < 0.001) (Figure 1). Compared to e-SIAAD, f-SIAA had a higher frequency of infrarenal primary entry, aortic arteriosclerosis, and penetrating aortic ulcer. In contrast, e-SIAAD had a bigger maximum dissection diameter and was more often associated with false lumen thrombosis and more involvement of the renal artery and common iliac artery.

### 4.3. Management and In-Hospital Outcomes

As shown in Table 3, management of SIAAD patients was medical in 48 (30.19%), endovascular in 104 (65.41%), and surgical in 4 (2.52%) patients. Initial medical therapy was administered with a β-blocker in 101 (63%) patients, an angiotensin-converting enzyme inhibitor or angiotensin receptor blocker in 55 (34%) patients, and a calcium channel blocker in 126 (79%) patients. Anti-platelet agents (aspirin and clopidogrel) and statins were more commonly used in f-SIAAD than in e-SIAAD.

There were 40 (25.16%) patients (12 f-SIAAD and 28 e-SIAAD) who had AAA. The abdominal aorta was replaced in four patients, two e-SIAAD, both of whom had aortic rupture, and two f-SIAAD had giant AAA (diameter > 70 mm). Notably, one f-SIAAD patient (man, 70 years) experienced severe aortic rupture immediately before surgery. Despite emergency replacement of the abdominal aorta under cardiopulmonary bypass, the patient remained hemodynamically unstable postoperatively and subsequently passed away. Overall, the in-hospital mortality rate, irrespective of e-SIAAD or f-SIAAD, was relatively low (7, 4%), with six f-SIAAD and one e-SIAAD cases. The mean hospital stay was 11.4 days, with 9 days for patients treated medically and 13 days for those undergoing surgery or endovascular repair. There were seven death cases in the hospital, among which six were treated medically and one with open surgery. The patient with open surgery underwent emergency abdominal aorta replacement for severe aortic rupture before surgery but remained unstable postoperatively and subsequently died. Of the six patients managed conservatively, two died from acute aortic rupture, for which their families refused surgery; one patient’s dissection involved the superior mesenteric artery (SMA), resulting in intestinal necrosis. This complication progressed to acute diffuse peritonitis and ultimately resulted in multiorgan failure and death. One patient was admitted for acute myocardial infarction (AMI) and died of heart failure during hospitalization. One patient admitted with acute liver failure during hospitalization developed severe pneumonia and respiratory failure, ultimately resulting in death. One patient admitted with advanced lung cancer also died during the hospital stay.

### 4.4. Long-Term Outcomes

**Figure 3:**
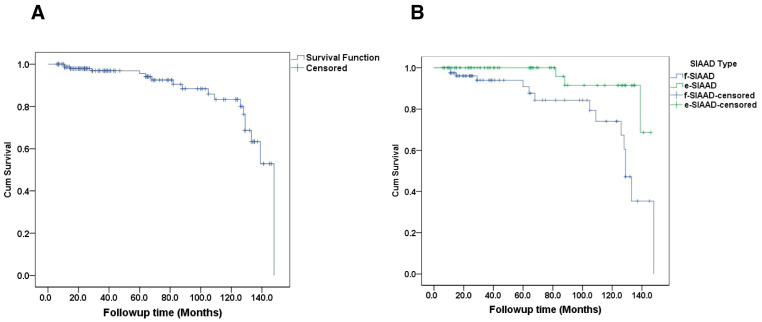
(**A**) Survival curve in patients with SIAAD. (**B**) the cumulative survival rate in focal-SIAAD is significantly lower than that in extensive-SIAAD (*p* = 0.010). SIAAD, spontaneous isolated abdominal aortic dissection.

After discharge, 11 patients died during follow-up and their detailed information is shown in Table 4. Mortality was reported in one patient treated surgically, one patient who underwent endovascular repair, and nine patients who received medical treatment. The median follow-up was 48 months (range, 6–148 months). The Kaplan–Meier estimated survival rate at 1 year was ~98%, at 5 years ~95%, and at 10 years ~80% for the entire cohort (Figure 3A). When stratified by dissection type, there was a notable divergence in survival curves (Figure 3B). Patients with f-SIAAD had lower survival over time than those with e-SIAAD. The estimated 5-year survival for f-SIAAD was approximately 78%, compared to ~95% for e-SIAAD. By 10 years, about 70% of f-SIAD patients were alive vs. ~90% of e-SIAAD (though few e-SIAAD had 10-year follow-up given many were younger at baseline).

Repeated aortic CTA was obtained in 56 (35%) of 159 patients during follow-up. Of 108 patients initially treated with surgery or endovascular repair, 42 had CTA follow-up, and all images showed a reduction in aortic diameter in the dissection segment. Of 48 patients initially treated with a conservative strategy, 14 (10 f-SIAAD and 4 e-SIAAD) had CTA follow-up. In the 10 f-SIAAD patients, the dissection image showed progressive enlargement in three (one patient had a giant AAA and refused surgery), improvement in one, and unchanged in six patients. In the four e-SIAAD patients, the dissection image showed progressive enlargement in one (late endovascular repair was scheduled for this patient) and improvement in three patients.

## 5. Discussion

The main findings of this study are as follows: (1) Patients with f-SIAAD were much older and had more cardiovascular risk factors (such as hypertension and diabetes mellitus), coronary artery disease, and chronic renal insufficiency than those with e-SIAAD; (2) Patients with e-SIAAD were more often admitted to the hospital in the acute phase and more frequently had SIAAD-related symptoms than those with f-SIAAD; (3) Aortic CTA showed that f-SIAAD was more often located at the infrarenal abdominal aorta and had arteriosclerosis and PAU.

In contrast, e-SIAAD more frequently had false lumen thrombosis and involved the renal and common iliac artery. Moreover, e-SIAAD also had wider and longer dissections; (4) The long-term prognosis in patients with f-SIAAD had a worse long-term survival than those with e-SIAAD, despite the focal dissections being anatomically smaller.

This retrospective study proposed a new morphological classification of SIAAD based on dissection length. As shown in Figure 1, a clear bimodal distribution emerges with natural separation near 50 mm, which we used as the cut-point for distinguishing f-SIAAD and e-SIAAD. In a previous study with detailed data on each patient, an apparent gap was observed in the ascending order of SIAAD length, with a mean dissection length of 54 mm [11]. From a procedural perspective, a segment of 5 cm is frequently referenced as the minimum length required for secure stent graft anchoring during endovascular repair, underscoring the practical significance of this threshold [12]. Importantly, when patients were grouped by this criterion, clear distinctions in both clinical presentation and anatomical features emerged, reinforcing the validity of this approach. Therefore, a cut-point of 50 mm may be reasonable.

The prevalence of SIAAD in our study aligns with previous studies, which range from 1% to 4% [1,13]. However, the true prevalence of SIAAD was likely underestimated, as a significant portion of patients in the study either presented without symptoms or had their dissection discovered incidentally. According to the International Registry of Acute Aortic Dissection (IRAD) study, up to 95% of patients experience dissection-related symptoms [2]. This could partly explain why less than half of the patients, 77 (48.43%), in this study were initially diagnosed with AD on admission. Moreover, compared to e-SIAAD, f-SIAAD patients were more likely to be admitted to the hospital in the chronic phase of AD and less likely to be initially diagnosed with AD on admission, mainly because more patients in f-SIAAD were asymptomatic than in e-SIAAD, suggesting that f-SIAAD is more likely to be missed than e-SIAAD.

SIAAD is often linked to hypertension, atherosclerosis, and preexisting aneurysmal changes [14,15]. In our updated data, AAA was present in 25% of cases. In addition, our data further expanded previous reports. Clinically, we observed that, compared with patients with e-SIAAD, those with f-SIAAD were older and more likely to have hypertension, diabetes mellitus, and coronary artery disease. Furthermore, aortic CTA image data showed that f-SIAAD had more aortic arteriosclerosis and PAU than e-SIAAD. Therefore, we believe that there might be some underlying pathology to explain these significant differences between f-SIAAD and e-SIAAD. A possible explanation might be that f-SIAAD is strongly associated with arteriosclerosis, and the presence of scarring and medial atrophy caused by arteriosclerosis limits the propagation of f-SIAAD. In addition to the length difference, we also found that, compared to e-SIAAD, the primary entry site of f-SIAAD was more commonly located in the infrarenal district. In contrast, e-SIAAD had a higher propensity for proximal entry: 37.8% of e-SIAAD had the primary entry tear above the renal arteries (suprarenal, involving the segment between the celiac axis and the lowest renal artery) compared to only 12.9% in f-SIAAD.

**Figure 4:**
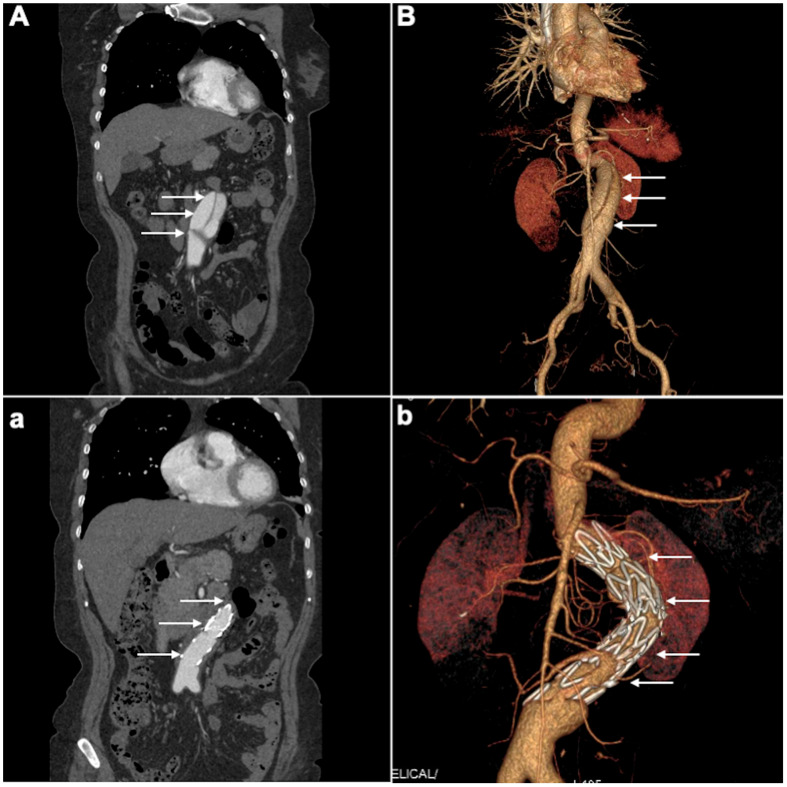
(**A**) pre-EVAR CTA intimal flap separates true and false lumens within the abdominal aorta. (**a**) post-EVAR CTA successful stent-graft coverage of entry tear with absence of false lumens opacification. (**B**) Pre-EVAR 3D-CTA visualizes the SIAAD extending through the infrarenal aorta with evidence of a dissecting aneurysm and a prominent false lumen. (**b**) Post-EVAR 3D-CTA complete exclusion of the false lumen and successful aneurysm repair, with the endovascular stent graft visible. White arrows denote the intimal flap, false lumen, and stent-graft deployment. 3D-CTA, Three-dimensional Computed Tomography Angiogram; SIAAD, spontaneous isolated abdominal aortic dissection.

Management for SIAAD includes medical treatment, endovascular repair, and surgery. In the IRAD study, medical therapy was performed in two-thirds of patients [2], whereas endovascular repair or surgery was adopted in most patients in other published studies. Treatments varied in different studies, and there is no available therapeutic guideline for SIAAD. Contrary to the IRAD findings, in this study, EVAR was the primary treatment in 65% of patients, making it the predominant strategy. This aligns with recent publications that report increasing use of EVAR for isolated abdominal dissections [16,17]. The rationale for EVAR in SIAAD is to exclude the entry tear and reinforce the dissected segment, thus preventing false lumen pressurization and potential aneurysm formation or rupture (Figure 4). Our results show that EVAR achieved excellent aortic remodeling in all treated patients, with no aorta-related deaths in that group. This finding echoes other reports, which have shown high success and low mortality rates for EVAR in SIAAD [16,18]. In fact, a recent systematic review found endovascular treatment of SIAAD to be associated with good long-term aortic remodeling and low complication rates [19].

**Figure 5:**
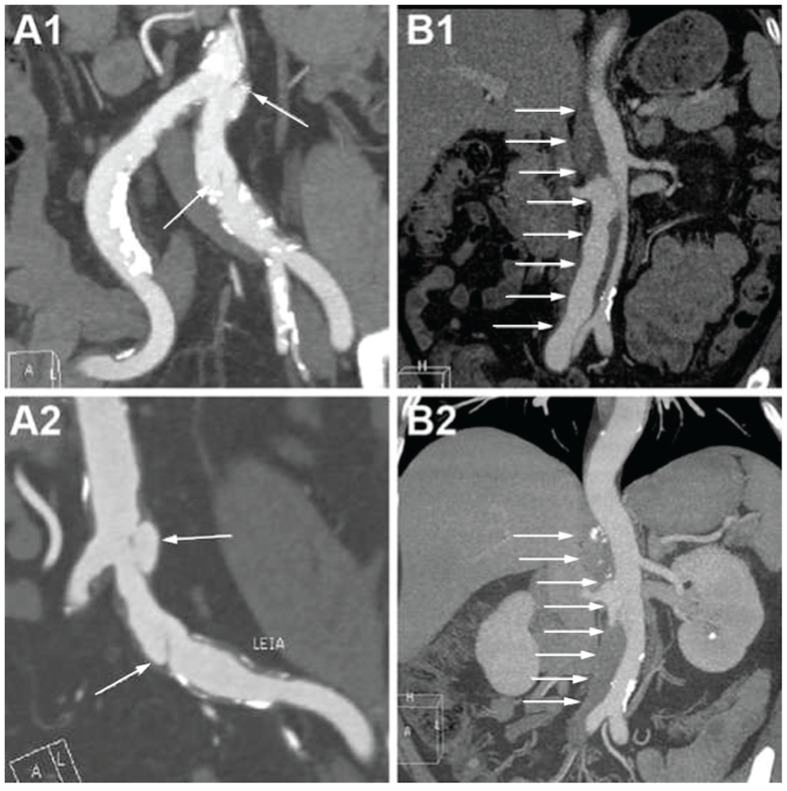
CTA images on admission and at follow-up in focal and extensive SIAAD. (**A1**) F-SIAAD on admission; (**A2**) F-SIAAD remained unchanged at 24 months after discharge; (**B1**) E-SIAAD on admission; (**B2**) the false lumen of E-SIAAD was almost completely thrombotic at 24 months after discharge. White arrows denote the intimal flap and false lumen. CTA, computed tomography angiography; SIAAD, spontaneous isolated abdominal aortic dissection.

Overall, the short- and long-term clinical outcomes of SIAAD were good. In our study, medical therapy was adopted in 30% of patients, endovascular therapy was performed in 65%, and surgical treatment was performed in 2.5%. Given the larger sample size of 159 patients and the extended follow-up period compared to previous smaller studies, clearer insights into therapeutic outcomes were possible. However, significant differences between treatment modalities remained limited. This reflects findings in the context of uncomplicated type B (thoracic) dissections, where randomized trials (e.g., ADSORB) showed improved aortic remodeling with TEVAR but no early survival difference [20]. The fact that about one-third of our patients were managed medically and did well indicates that not every SIAAD requires stenting. For the f-SIAAD patients, 15 patients died in the hospital and the follow-up period, while only 4 were aorta-related deaths. Additionally, patients with f-SIAAD were frequently associated with old age, more co-morbidities, and fewer SIAAD-related symptoms. Hence, strict medical management focusing on the control of risk factors, heart rate, and blood pressure is advisable as a suitable strategy for f-SIAAD patients. For the e-SIAAD patients, only three patients died in hospital and during the follow-up period. Of these, one was attributed to an aorta-related cause, while the causes of the remaining two deaths were unknown. Additionally, the rate of early outcome for false lumen thrombosis was relatively high (41%), with some cases even being completely thrombotic (Figure 5(B1,B2)). Therefore, it is suggested that, in uncomplicated e-SIAAD patients, conservative treatment can occasionally be appropriate. Still, given the lack of reliable predictors of which will enlarge, ongoing surveillance to identify signs of dissection, AAA progression, and rupture is prudent for both f-SIAAD and e-SIAAD patients.

Interestingly, although the dissection length was much shorter in f-SIAAD than e-SIAAD, the long-term clinical follow-up tended to be worse in f-SIAAD. There are several reasons behind this “paradoxical” phenomenon. First, patients with f-SIAAD were much older and had more cardiovascular risk factors (like hypertension and diabetes) than those with e-SIAAD. Second, f-SIAAD was more often associated with arteriosclerosis and coronary artery disease. Third, f-SIAAD patients more frequently had chronic renal insufficiency than e-SIAAD patients. Hence, the life expectancy was speculated to be “paradoxically” shorter in f-SIAAD than e-SIAAD.

In conclusion, this study introduces a novel morphological classification of SIAAD into two subtypes: focal (f-SIAAD) and extensive (e-SIAAD), demonstrating significant prognostic and therapeutic implications. While our findings support the clinical utility of this classification, further prospective multicenter research is required to validate its predictive value and refine management strategies.

## 6. Conclusions

SIAAD may be further divided into focal and extensive SIAAD according to the dissection length. The significant distinctions in age, risk factors, clinical presentations, and CTA features between incident arteriosclerosis and vascular conditions in e-SIAAD and f-SIAAD indicate possible different mechanisms underlying them. Despite these differences, prompt diagnosis and appropriate management are key to good outcomes in both groups.

Endovascular aortic repair has become the first-line treatment for many patients, reflecting a shift in practice and offering excellent aortic remodeling. Conservative management remains a valid approach in select cases; however, careful monitoring is necessary to detect any late aortic enlargement. For selected patients with uncomplicated e-SIAAD, conservative management with close surveillance to identify signs of disease progression is justifiable. However, in cases of complicated e-SIAAD or high-risk anatomical features, endovascular intervention is now widely favored due to its safety, efficacy, and favorable impact on aortic remodeling, making it the preferred treatment modality in current practice. Moreover, the overall response to medical therapy in SIAAD was positive regardless of the trend that f-SIAAD may result in a worse long-term clinical prognosis than e-SIAAD. Ongoing research and collaboration will further refine these strategies, leading to the development of formal guidelines to assist in the care of patients with this uncommon form of AD.

## 7. Limitations

There are several limitations in our study. First, this study was observational and retrospective, conducted at a single high-volume center, which may introduce selection bias in patient inclusion and management decisions. Second, we cannot compare outcomes based on treatment approach in the two groups because of the same retrospective design reason. Ideally, patients with SIAAD would be randomized in a trial to analyze the results of the different treatment modalities. Third, due to the study’s nature, aortic CTA was not performed routinely during follow-up, and repeated CTA images were obtained in only a percentage of patients. Therefore, there is a bias in evaluating aortic remodeling in patients with SIAAD due to the limited data available. Fourth, while our sample size is substantial for this rare condition, the low frequency of critical outcome events (e.g., deaths, aortic ruptures, re-interventions) may reduce the statistical power of subgroup analyses, increasing the risk that observed differences may reflect random variation rather than true associations. Fifth, dissection length was not indexed to patient size, which may introduce anatomical bias, as the clinical implications of a 50 mm dissection may vary according to individual patient anatomy. Future studies should normalize measurements to improve classification accuracy. Finally, as f-SIAAD is strongly associated with aortic arteriosclerosis and PAU, it is often difficult to clearly distinguish between f-SIAAD and “intra-plaque dissection” caused by plaque rupture or PAU expansion based on imaging alone; as long as the “intra-plaque dissection” satisfies the definition of f-SIAAD (intimal flap length ≤ 50 mm and true/false lumens), we classified it as f-SIAAD. Finally, our results and proposed classification threshold require validation in larger, prospective, multicenter studies before they can be widely adopted in clinical practice.

## Figures and Tables

**Table 1:** Baseline characteristics of SIAAD according to the dissection length.

		Total	f-SIAAD	e-SIAAD	*p*-Value
		(*n* = 159)	(*n* = 85)	(*n* = 74)	
Age		57.7 ± 12.4	63.74 ± 10.97	50.70 ± 10.10	0.000
Sex	Female	40 (25.16)	21 (24.71)	19 (25.68)	0.888
	Male	119 (74.84)	64 (75.29)	55 (74.32)	
Smoking	No	109 (68.55)	61 (71.76)	48 (64.86)	0.350
	Yes	50 (31.45)	24 (28.24)	26 (35.14)	
Alcohol	No	124 (77.99)	64 (75.29)	60 (81.08)	0.380
	Yes	35 (22.01)	21 (24.71)	14 (18.92)	
Hypertension	No	47 (29.56)	18 (21.18)	29 (39.19)	0.013
	Yes	112 (70.44)	67 (78.82)	45 (60.81)	
Diabetes mellitus	No	132 (83.02)	60 (70.59)	72 (97.3)	0.000
	Yes	27 (16.98)	25 (29.41)	2 (2.7)	
Hyperlipidemia	No	128 (80.5)	62 (72.94)	66 (89.19)	0.010
	Yes	31 (19.5)	23 (27.06)	8 (10.81)	
Chronic renal insufficiency	No	133 (83.65)	66 (77.65)	67 (90.54)	0.028
	Yes	26 (16.35)	19 (22.35)	7 (9.46)	
Coronary artery disease	No	103 (64.78)	38 (44.71)	65 (87.84)	0.000
	Yes	56 (35.22)	47 (55.29)	9 (12.16)	
COPD	No	154 (96.86)	81 (95.29)	73 (98.65)	0.227
	Yes	5 (3.14)	4 (4.71)	1 (1.35)	
SIAAD-related symptoms	No	44 (27.67)	33 (38.82)	11 (14.86)	0.0037
	Yes	115 (72.33)	52 (61.18)	63 (85.14)	
Chest Pain	No	115 (72.33)	65 (76.47)	50 (67.57)	0.211
	Yes	44 (27.67)	20 (23.53)	24 (32.43)	
Abdominal Pain	No	97 (61.01)	59 (69.41)	38 (51.35)	0.020
	Yes	62 (38.99)	26 (30.59)	36 (48.65)	
Back Pain N	No	128 (80.5)	72 (84.71)	56 (75.68)	0.152
	Yes	31 (19.5)	13 (15.29)	18 (24.32)	
Lumbar Pain	No	132 (83.02)	77 (90.59)	55 (74.32)	0.006
	Yes	27 (16.98)	8 (9.41)	19 (25.68)	
Acute chronicity					0.000
Acute-SIAAD		83 (52.2)	29 (34.12)	54 (72.97)	
Chronic-SIAAD		76 (47.8)	56 (65.88)	20 (27.03)	
Dissection as initial diagnosis on admission	No	82 (51.5)	64 (75.29)	23 (31.08)	0.000
	Yes	77 (48.43)	21 (24.71)	51 (68.92)	

Values are mean ± SD or *n* (%). SIAAD, spontaneous isolated abdominal aortic dissection.

**Table 2:** Aortic CTA findings of SIAAD according to the dissection length.

		Total	f-SIAAD	e-SIAAD	*p*-Value
		(*n* = 159)	(*n* = 85)	(*n* = 74)	
Location of the primary entry site					
Supraceliac	No	120 (75.47)	74 (87.06)	46 (62.16)	0.000
	Yes	39 (24.53)	11 (12.94)	28 (37.84)	
Paravisceral	No	142 (89.31)	80 (94.12)	62 (83.78)	0.035
	Yes	17 (10.69)	5 (5.88)	12 (16.22)	
Infrarenal	No	56 (35.22)	16 (18.82)	40 (54.05)	0.000
	Yes	103 (64.78)	69 (81.18)	34 (45.95)	
SIAAD length, mm		68.44 ± 56.37	24.48 ± 10.92	118.93 ± 43.65	0.000
SIAAD maximum diameter, mm		27.89 ± 11.72	24.41 ± 11.28	31.89 ± 10.99	0.000
Involvement					
Celiac trunk artery	No	152 (95.6)	80 (94.12)	72 (97.3)	0.330
	Yes	7 (4.4)	5 (5.88)	2 (2.7)	
Superior mesenteric artery	No	155 (97.48)	83 (97.65)	72 (97.3)	0.888
	Yes	4 (2.52)	2 (2.35)	2 (2.7)	
Renal artery	No	127 (79.87)	75 (88.24)	52 (70.27)	0.005
	Yes	32 (20.13)	10 (11.76)	22 (29.73)	
Inferior mesenteric artery	No	137 (86.71)	80 (95.24)	57 (77.03)	0.001
	Yes	21 (13.29)	4 (4.76)	17 (22.97)	
Common iliac artery	No	79 (49.69)	63 (74.12)	16 (21.62)	0.000
	Yes	80 (50.31)	22 (25.88)	58 (78.38)	
False lumen thrombosis	No	117 (73.58)	74 (87.05)	43 (58.10)	0.0002
	Yes	42 (26.41)	11 (12.94)	31 (41.89)	
Arteriosclerosis	No	81 (50.94)	24 (28.24)	57 (77.03)	0.000
	Yes	78 (49.06)	61 (71.76)	17 (22.97)	
AAA	No	119 (74.84)	73 (85.88)	46 (62.16)	0.001
	Yes	40 (25.16)	12 (14.12)	28 (37.84)	
PAU	No	110 (69.18)	47 (55.29)	63 (85.14)	0.000
	Yes	49 (30.82)	38 (44.71)	11 (14.86)	
Pleural effusion	No	122 (76.73)	71 (83.53)	51 (68.92)	0.030
	Yes	37 (23.27)	14 (16.47)	23 (31.08)	

Values are mean ± SD or *n* (%). SIAAD, spontaneous isolated abdominal aortic dissection; CTA, computed tomography angiography.

**Table 3:** In-hospital management and outcome of SIAAD according to the dissection length.

		Total	f-SIAAD	e-SIAAD	*p*-Value
		(*n* = 159)	(*n* = 85)	(*n* = 74)	
In-hospital treatment					
Medication	No	108 (67.92)	53 (62.35)	55 (74.32)	0.272
	Yes	48 (30.19)	29 (34.12)	19 (25.68)	
Endovascular repair	No	55 (34.59)	34 (40)	21 (28.38)	0.307
	Yes	104 (65.41)	51 (60)	53 (71.62)	
Open surgery	No	155 (97.48)	83 (97.65)	72 (97.3)	0.99
	Yes	4 (2.52)	2 (2.35)	2 (2.7)	
In-hospital medical therapy					
Aspirin	No	126 (79.25)	60 (70.59)	66 (89.19)	0.004
	Yes	33 (20.75)	25 (29.41)	8 (10.81)	
Clopidogrel	No	141 (88.68)	70 (82.35)	71 (95.95)	0.007
	Yes	18 (11.32)	15 (17.65)	3 (4.05)	
Statins	No	89 (55.97)	41 (48.24)	48 (64.86)	0.035
	Yes	70 (44.03)	44 (51.76)	26 (35.14)	
βblocker	No	58 (36.48)	31 (36.47)	27 (36.49)	0.998
	Yes	101 (63.52)	54 (63.53)	47 (63.51)	
CCB	No	33 (20.75)	18 (21.18)	15 (20.27)	0.888
	Yes	126 (79.25)	67 (78.82)	59 (79.73)	
ACEI/ARB	No	104 (65.41)	52 (61.18)	52 (70.27)	0.229
	Yes	55 (34.59)	33 (38.82)	22 (29.73)	
In-hospital outcome					
Hospitalization time		11.4 ± 8	12 ± 8	11 ± 6	0.819
In-hospital mortality	No	152 (95.60)	79 (92.94)	73 (98.65)	0.08
	Yes	7 (4.40)	6 (7.06)	1 (1.35)	

Values are mean ± SD or *n* (%). SIAAD, spontaneous isolated abdominal aortic dissection; ACEI, angiotensin-converting enzyme inhibitor; ARB, angiotensin receptor blocker; CCB, calcium channel blocker.

**Table 4:** Information of death cases during hospitalization and follow-up.

Patient Number	Sex/Age	SIAAD Type	SIAAD-Related Symptoms	Acute Chronicity	Cardiovascular Risk Factor	AAA	Comorbidity	Treatment	Follow-Up (Months)	Time of Death Month	Death Cause
1	M/67	Extensive	Chest pain	Chronic	CAD	No	No	Endovascular Repair	88	Unknown	Unknown
2	M/68	Focal	Abdominal pain	Chronic	HTN, CAD, Smo	No	COPD, DLBCL	Conservative	29	28	Respiratory failure
3	M/43	Extensive	Abdominal pain, back pain	Acute	No	No	No	Conservative	In-hospital mortality	In-hospital mortality	Acute aortic rupture
4	M/82	Focal	Asymptomatic	Chronic	HTN, HL, CAD, Smo	No	AMI	Conservative	In-hospital mortality	In-hospital mortality	Heart failure
5	M/66	Focal	Asymptomatic	Chronic	CAD	Yes	CRI	Conservative	In-hospital mortality	In-hospital mortality	Advanced lung cancer
6	M/75	Focal	Asymptomatic	Chronic	CAD, COPD, Smo	No	HCC	Conservative	15	8	Liver cancer
7	F/59	Focal	Abdominal pain	Chronic	DM	No	No	Conservative	In-hospital mortality	In-hospital mortality	Acute liver failure
8	M/70	Focal	Abdominal pain	Acute	HTN	Yes	CRI	Open Surgery	In-hospital mortality	In-hospital mortality	AAA rupture
9	M/72	Focal	Back pain	Chronic	HTN, CAD	Yes	MI	Open Surgery	129	Unknown	Unknown
10	M/73	Focal	Asymptomatic	Chronic	HTN	No	CRI	Conservative	133	31	Unknown
11	M/85	Focal	Asymptomatic	Chronic	HTN, HL, DM, Smo	No	AI	Conservative	129	11	Heart failure
12	M/69	Focal	Chest pain	Chronic	HTN, CAD	No	AMI	Conservative	11	11	re-AMI
13	M/77	Focal	Abdominal pain	Acute	HTN, CAD	No	No	Conservative	In-hospital mortality	In-hospital mortality	SMA dissection leading to multiorgan failure
14	M/55	Focal	Chest pain, back pain	Acute	HTN	Yes	No	Conservative	109	4	AAA rupture
15	M/71	Focal	Abdominal pain	Acute	HTN	Yes	RAI	Conservative	In-hospital mortality	In-hospital mortality	AAA rupture
16	M/65	Focal	Back pain	Acute	HTN, DM, CAD, Smo	No	No	Conservative	128	34	Unknown
17	F/70	Focal	Abdominal pain, chest pain	Chronic	HTN, CAD, COPD	Yes	No	Conservative	148	32	Unknown
18	M/52	Extensive	Abdominal pain, chest pain	Acute	HTN	Yes	COPD	Conservative	82	41	Unknown

AAA, abdominal aortic aneurysm; AMI, acute myocardial infarction; CAD, coronary artery disease; CRI, chronic renal insufficiency; DM, diabetes mellitus; HL, hyperlipidemia; HTN, hypertension; Smo, Smoking; SMA, superior mesenteric artery; COPD, chronic obstructive pulmonary disease; DLBCL, diffuse large B cell lymphoma; AI, aortic insufficiency; HCC, hepatocellular carcinoma; MI, mitral incompetence; RAI, Rheumatic aortic insufficiency.

## Data Availability

The datasets used and analyzed during the current study are available from the corresponding author upon reasonable request.
